# Multiple Subcutaneous Calcifications in a Neonate after Intravenous Calcium Gluconate: A Case Report

**DOI:** 10.31729/jnma.v63i290.9197

**Published:** 2025-09-01

**Authors:** Anjila Ghimire

**Affiliations:** 1 Department of Pediatrics, Kathmandu Medical College teaching hospital, Kathmandu, Nepal

**Keywords:** *extravasation*, *hypocalcemia*, *iatrogenic calcinosis cutis*, *soft tissue calcification*

## Abstract

Extravasation of calcium gluconate can lead to erythema, tenderness, induration, edema and sometimes iatrogenic calcinosis cutis. Subcutaneous calcification may be mistaken for cellulitis, abscess, calcified hematoma, or osteomyelitis, resulting in unnecessary antibiotic therapy or surgical intervention. A 36+5 weeks male baby with birth weight 2500 grams, presented on 4th day of life with fever and neonatal hyperbilirubinemia. He developed symptomatic hypocalcemia and was treated with multiple doses of intravenous calcium gluconate infusion. At one week follow up he presented with multiple tender hard nodules distributed over previous cannulation sites. He was managed conservatively. Paracetamol was given for pain management. There was complete resolution of lesions by 12 weeks. Radiographs in soft tissue calcification after extravasation of calcium gluconate are initially negative, take one to three weeks to appear and usually months to resolve.

## INTRODUCTION

Hypocalcemia is treated by intravenous slow infusion of 10% calcium gluconate via a separate peripheral venous access in the dose of 1-2 ml/kg/dose.^1^ Extravasation of the drug can result in erythema, subcutaneous calcification, tissue necrosis, skin slough and transient radial nerve damage.^2^ Subcutaneous calcifications occur as firm subcutaneous nodules or areas of inflammation with central softening and fluctuation.^3^ They are associated with evidence of calcification in the X-ray. However, clinically they can be confused with cellulitis, abscess or osteomyelitis, and managed with unnecessary medical or surgical intervention where conservative management is sufficient.

## CASE REPORT

A male infant was born at 36 weeks 5 days with birth weight of 2500 grams to a primi gravida mother without any known comorbidity via spontaneous vaginal delivery was admitted to our hospital on 4^th^ day of life with complaint of yellowish discoloration of body for 2 days associated with decreased feeding, fever and decreased urine output. Baby was under mixed feeding till that period. Lab reports showed unconjugated hyperbilirubinemia in exchange range (19.9 mg/dl) with low values of total and ionized calcium (6.5 mg/dl, 3.2mg/dl) in addition to clinical features suggestive of early onset neonatal sepsis like fever, lethargy, poor feeding and respiratory distress in the form of tachypnea, cyanosis and mild subcostal retractions. Along with management of neonatal sepsis as per the unit protocol, we started intensive phototherapy. The chest x-ray was within normal limits. Clinically, there was jitteriness as a sign of hypocalcemia.

**Figure:**
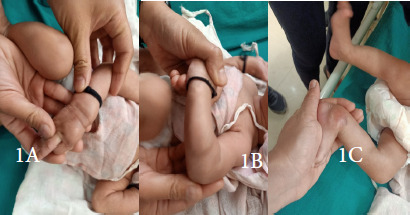
**Image 1A:** Hard lump over dorsum of hand; **Image 1B:** Hard lump over dorsum of forearm; **Image 1C:** Hard lump over the ankle.

We treated the baby with intravenous 10% Calcium gluconate at the rate of 400 mg/kg day (4 ml/kg/day). The calcium values normalized after almost 72 hours of continuous infusion. When switched to oral Calcium maintenance at 50 mg/kg/ day, there was secondary decline in calcium level so we had to supplement intravenous calcium for another 48 hours before switching to oral supplement. During workup, he had Vitamin D insufficiency (25-hydroxy cholecalciferol= 24.73 ng/ml) with normal intact Parathyroid hormone level (iPTH= 30.7pg/ml), normal Alkaline phosphatase (143 IU/L) and normal phosphorus level (7.9 mg/dl). The cerebrospinal fluid analysis done showed normal reports with no cells, normal gram stain and sterile CSF culture. Blood culture was sterile. Cranial ultrasound was normal. Fever subsided after 48 hours of admission, respiratory distress subsided and baby was off oxygen by day five of admission. Baby was initiated feeds next day of admission with orogastric feeds, feeding was rapidly escalated to full feeds and he started breastfeeding from day five of admission. Baby was discharged after 15 days of admission with the diagnosis of early onset culture sterile neonatal sepsis with clinically suspected meningitis with sterile CSF with neonatal hyperbilirubinemia with ABO setting (mother’s blood group: O positive, baby’s: B positive) with hypocalcemia with vitamin D insufficiency. Neurological examination was normal.

**Figure:**
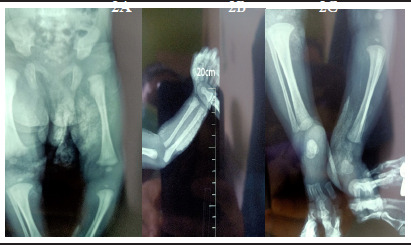
**Image 2A:** X-ray image showing soft tissue calcification over the thighs, **Image 2B:** X-ray showing linear calcification over the forearm, **Image 2C:** X-ray image showing calcification over the ankle

At the time of discharge baby had erythematous soft tissue swelling at cannula site on dorsum of right hand which we treated as thrombophlebitis. At one week follow up baby was brought with tender bony-hard swellings over different parts of the upper and lower limbs at sites of previous cannulations with erythematous overlying skin. However, there was no fluctuation or soft tissue swelling or pus point or pus discharge from any site which clinically excluded cellulitis and abscess.

**Figure:**
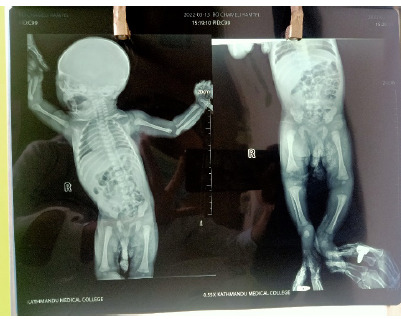
**Image 3.** Infantogram showing multiple areas of soft tissue calcification

We did X-ray of all the involved sites of limbs which showed calcification in the soft tissues without extension to the underlying bones which ruled out osteomyelitis. We requested the parents for biopsy and ultrasound of affected sites but they declined. Conservative management was done with oral analgesics (Paracetamol) and avoidance of massage at the involved sites. There was gradual resolution of the lesions on follow up and they disappeared completely after 12 weeks.

## DISCUSSION

Skin and soft tissue calcification after intravenous calcium gluconate infusion in newborn is a distressing clinical entity. The presentation ranges from skin erythema and discoloration, necrosis, cellulitis, skin and soft tissue calcifications to superficial nerve damage and compartment syndrome.^4^ Extravasation refers to the leakage of an injected substance from the blood vessels, leading to its flow into the surrounding tissues.^5^ Acute symptoms associated with extravasation like irritability, swelling, erythema, vesicle formation, cellulitis, skin discoloration and necrosis appear early. Calcifications of skin and soft tissues takes days to weeks for appearance.^6^ Infants present with fever, pain, inflammation, and acute tenderness in the limb leading towards a diagnosis of acute infection.^7^ These lesions are usually tender so babies become irritable when handled. Relevant history, x-rays and biopsy of lesion from the affected site usually establishes the diagnosis.

Case report published by J R Roberts stated a rare event of radial nerve damage with wrist drop because of extravasation of calcium gluconate into the subcutaneous tissues. However, most cases present with milder forms of acute symptoms. Acute symptoms associated with extravasation are-irritability, swelling, erythema, vesicle formation, cellulitis, skin discoloration and necrosis. Calcification of skin and soft tissues however, take days to weeks for appearance. Damaged subcutaneous tissue and cell necrosis at the extravasation site create an acidic environment which lacks calcification inhibitors thus facilitating calcification.^8^ A case of iatrogenic calcinosis cutis with distant and delayed extravasation of calcium gluconate over the flank of neonate who received IV calcium gluconate via the femoral vein has also been described.^9^

In our case there was appearance of hard, tender, nodular lumps over the previous cannulation sites about one week after discharge from the hospital. Considering a hospital stay of 14 days, the lesions had evolved and appeared about 1-3 weeks after the extravasation. At follow up these lesions appeared like osteomyelitis because there was erythema of skin, irritability and bony swellings. As there were multiple lesions, X-ray was done. Presence of subcutaneous calcifications along the previous cannulation sites without extension to the underlying bones and absence of periosteal reaction confirmed the diagnosis of iatrogenic subcutaneous calcification following calcium gluconate injection.

Intralesional steroid infiltration, diltiazem, bisphosphonates and surgical interventions to reduce local signs have been proposed as treatments for calcification of tissues however, minor lesions usually undergo self-resolution over a period of weeks to months.^10^ In our patient, the palpable calcification lesions disappeared completely after 12 weeks however, we couldn’t get a radiological confirmation as the visitors denied further investigation.

## CONCLUSION

Extravasation of calcium gluconate can give rise to hard lumps of subcutaneous calcification creating a diagnostic dilemma so a careful review of history, clinical findings and X-ray images is essential to avoid unnecessary medical or surgical intervention.
